# Transformer-based ensemble framework for tuberculosis treatment response prediction: multi-omics integration with clinical-grade performance

**DOI:** 10.3389/fmolb.2026.1788211

**Published:** 2026-05-20

**Authors:** Huanhuan Ba, Huanqing Liu, Tingting Li, Juan Jin

**Affiliations:** 1 Department of Infectious Diseases, The Eighth’s Hospital of Xi’an, Xi’an, Shaanxi, China; 2 Information Management Office, Northwestern Polytechnical University, Xi’an, Shaanxi, China; 3 Drug Clinical Trial Institution Office, Xi’an Chest Hospital, Xi’an, Shaanxi, China

**Keywords:** deep learning, ensemble learning, multi-omics integration, transformer, treatment response prediction, tuberculosis

## Abstract

**Background:**

Tuberculosis (TB) treatment response prediction is critical for personalized care. We developed an ensemble Transformer framework integrating clinical and transcriptomic data from 467 patients and five GEO datasets (GSE83456, GSE107995, GSE158802, GSE19435, GSE25534).

**Methods:**

Five diverse Transformer architectures were trained with Focal Loss, label smoothing, and stratified 5-fold cross-validation. Outcome labels were harmonized using sputum culture conversion at 2 months.

**Results:**

The model achieved 97.1% accuracy and AUC 0.949 [95% CI: 0.912–0.978] on independent test folds, with sensitivity and specificity both 95%. Bootstrap validation (n = 1000) confirmed robustness.

**Conclusion:**

This framework provides a clinically-relevant tool for TB treatment response prediction, pending prospective validation.

**Clinical Trial Registration:**

https://www.chictr.org.cn/, identifier ChiCTR2300074328 03/08/2023.

## Introduction

Tuberculosis (TB), caused by *Mycobacterium tuberculosis*, remains a leading infectious cause of mortality worldwide. According to the World Health Organization, it is responsible for an estimated 10 million new cases and 1.5 million deaths annually (World Health Organization, 2022). Despite the existence of potent anti-tuberculosis drugs, treatment outcomes vary substantially among individuals. This heterogeneity is influenced by a complex interplay of factors, including bacterial drug resistance profiles, the host’s immune response, human genetic polymorphisms, and underlying comorbidities, all of which can significantly modulate therapeutic efficacy (Goletti et al., 2018). The capacity to accurately predict treatment response early during the course of therapy is crucial. Such prediction would facilitate personalized treatment regimens, with the potential to enhance clinical outcomes, minimize drug-related adverse effects, and optimize healthcare resource utilization (Imperial et al., 2018).

Recent advances in high-throughput technologies have yielded vast amounts of multi-omics data—encompassing genomics, transcriptomics, proteomics, and comprehensive clinical parameters. The integration of these heterogeneous data sources presents unprecedented opportunities for discovery alongside significant analytical challenges (Picard et al., 2021). Conventional statistical methods are often inadequate for capturing the complex, non-linear relationships and high-order interactions inherent across diverse data modalities (Zitnik et al., 2019). In contrast, deep learning approaches, particularly Transformer architectures initially developed for natural language processing, have demonstrated remarkable success across multiple domains. These models are increasingly being applied to biological data analysis due to their capacity to model long-range dependencies and capture intricate patterns through self-attention mechanisms (Eraslan et al., 2019).

Transformer models leverage self-attention mechanisms to dynamically assign importance weights to different input features. This capability renders them particularly well-suited for multi-omics data integration, where the biological relevance of individual features can vary significantly depending on the specific cellular or pathological context (Li et al., 2019). Furthermore, ensemble learning approaches, which aggregate predictions from multiple base models, have consistently demonstrated superior performance over single-model frameworks. By mitigating model variance and enhancing robustness, ensemble methods effectively improve predictive generalization on complex biomedical datasets (Walsh et al., 2021).

In this study, we presented an ultra-optimized ensemble Transformer framework that integrated clinical data and transcriptomic profiles to predict TB treatment response with clinical-grade accuracy (≥95%). Our approach combined multiple advanced techniques including deep Transformer architectures, ensemble learning with diverse model configurations, data augmentation, sophisticated loss functions, and rigorous statistical validation. We demonstrated that this integrated approach not only achieved exceptional predictive performance but also provided interpretable insights through attention visualization and pathway enrichment analysis. This work represented a significant step toward precision medicine in TB management and demonstrated the potential of advanced deep learning architectures in clinical decision support systems.

The key novelties of this work are: (1) the first ultra-optimized ensemble Transformer framework specifically designed for TB treatment response prediction; (2) rigorous integration of heterogeneous GEO datasets with explicit harmonization and dataset-level stratification to prevent data leakage; (3) achievement of ≥95% accuracy with comprehensive statistical validation including bootstrap confidence intervals and paired t-tests; and (4) multi-level interpretability via SHAP, LIME, and pathway enrichment analysis, linking model predictions to underlying biological mechanisms for clinical transparency. These innovations collectively address critical gaps in existing TB prediction models and establish a new benchmark for clinical-grade performance.

## Methods

### Clinical data collection

Clinical data were retrospectively collected from an institutional database of Xi’an Chest Hospital, covering 467 tuberculosis patients treated between September 2023 and December 2024. The study protocol was approved by the Institutional Review Board (S2023-0002) and conducted in accordance with the Helsinki Declaration. The dataset comprised 34 clinically relevant variables spanning multiple domains: (1) demographic characteristics (age, gender, body mass index (BMI)); (2) lifestyle factors (smoking status, alcohol consumption); (3) treatment parameters (isoniazid (INH) and rifampin (RFP) dosing, intervention group assignment); (4) hematological parameters (complete blood count with differential); (5) liver function tests (bilirubin, albumin, ALT, AST, ALP); (6) renal function markers (creatinine clearance, serum creatinine, uric acid); (7) lipid profile (total cholesterol, triglycerides); (8) inflammatory markers (C-reactive protein (CRP), erythrocyte sedimentation rate (ESR), procalcitonin); (9) immunologic parameters (CD4^+^/CD8^+^ T-cell percentages, T-SPOT. TB results); and (10) clinical outcomes (length of stay, adverse events, co-diagnoses, sputum smear status). Missing values were imputed using median imputation for continuous variables, and categorical variables were encoded using label encoding. All continuous features were standardized using StandardScaler to ensure comparable scales across different measurement units.

All immunological and inflammatory markers (CRP, ESR, procalcitonin, CD4+/CD8+ T-cell percentages) were obtained from venous blood draws at baseline within 72 h of treatment initiation. Heterogeneous timepoints were excluded from the primary analysis.

Cohort composition by drug resistance status (Table 1): drug-susceptible TB (DS-TB) 382 patients (81.8%), multidrug-resistant TB (MDR-TB) 58 patients (12.4%), pre-extensively drug-resistant TB (pre-XDR-TB) 18 patients (3.9%), and mono-resistant TB 9 patients (1.9%). DS-TB patients received standard first-line therapy (2HRZE/4HR); MDR-TB patients received WHO-recommended longer regimens.

**TABLE 1:** Cohort stratification by drug resistance status.

Category	N	%
Drug-susceptible TB (DS-TB)	382	81.8
Multidrug-resistant TB (MDR-TB)	58	12.4
Pre-extensively drug-resistant (pre-XDR-TB)	18	3.9
Mono-resistant TB	9	1.9
Total	467	100

Table 1 presents cohort stratification by drug resistance status. The majority of patients (81.8%, n = 382) had drug-susceptible TB (DS-TB), consistent with the epidemiologic profile in China. MDR-TB and pre-XDR-TB comprised 12.4% and 3.9% respectively, reflecting inclusion of challenging subgroups. This stratification ensures the model was trained on a clinically representative spectrum, enhancing generalizability to real-world settings where resistance status influences treatment selection and outcome prediction.

### Transcriptomics data integration

#### Batch effect correction

To address potential batch effects arising from the integration of multiple GEO datasets, we applied ComBat, a widely-used empirical Bayes method for batch effect correction, which adjusts for known batches while preserving biological variation. The ComBat algorithm models batch effects as additive and multiplicative terms, allowing for dataset-specific adjustments. We verified the effectiveness of batch correction by calculating the percentage of variance explained by batch before and after correction using ANOVA, ensuring that batch effects were reduced while biological signal was preserved. The corrected datasets showed <5% variance explained by batch (compared to 15%–25% before correction), indicating successful batch effect removal.

#### Feature selection strategy

Feature selection was performed in a multi-stage process to ensure both statistical significance and biological relevance. For transcriptomics data, we first identified highly variable genes (HVGs) using the coefficient of variation (CV) method, selecting the top 3000 genes with CV > 0.5. We then applied univariate statistical tests (Mann-Whitney U test for binary comparisons) to identify differentially expressed genes (DEGs) with false discovery rate (FDR) < 0.05. Additionally, we incorporated pathway-based feature selection by prioritizing genes within tuberculosis-related KEGG pathways (hsa05152, hsa04612, hsa04064). For clinical features, we performed correlation analysis to remove highly correlated features (Pearson correlation >0.9), retaining the most clinically interpretable variable. We also applied recursive feature elimination (RFE) with cross-validation to identify the optimal feature subset. The final feature set consisted of 34 clinical features and 2000 transcriptomic features, selected based on a combination of statistical significance, biological relevance, and model performance.

### Data normalization and quality control

All transcriptomics datasets underwent rigorous quality control and normalization procedures. Raw expression values were log2-transformed after adding a pseudocount of one to stabilize variance. We then applied quantile normalization to ensure comparable distributions across datasets. Quality metrics including RNA integrity, sequencing depth, and sample clustering were assessed. Missing values in clinical data were imputed using median imputation for continuous variables and mode imputation for categorical variables, with <5% missingness across all features. All continuous clinical features were standardized using StandardScaler (z-score normalization) to ensure comparable scales.

We integrated transcriptomics data from five publicly available Gene Expression Omnibus (GEO) datasets: GSE83456 (120 samples: TB patients vs. healthy controls), GSE107995 (96 samples: TB immune response), GSE158802 (85 samples: TB drug resistance), GSE19435 (150 samples: TB gene expression profiles), and GSE25534 (78 samples: TB proteomics-related). Expression data were log2-transformed to stabilize variance, normalized using quantile normalization, and the top 3000 most variable genes were selected for downstream analysis to reduce dimensionality while preserving biological signal. Batch effects were mitigated through careful normalization procedures.

GEO Dataset Harmonization and Outcome Mapping. Outcome labels were harmonized as follows: GSE83456 and GSE19435—outcomes mapped to treatment-naïve vs. treated; GSE107995 and GSE25534—treatment response labels used where available; GSE158802—included as supplementary feature source. Training and testing used strict dataset-level stratification: 80% from each dataset for training, 20% held out, preventing information leakage.

### Ultra-optimized model architecture

Our ensemble model consists of five diverse Transformer architectures with different configurations to ensure model diversity: (1) Model 1: d_model = 512, num_heads = 8, num_layers = 6; (2) Model 2: d_model = 768, num_heads = 12, num_layers = 8; (3) Model 3: d_model = 1024, num_heads = 16, num_layers = 6; (4) Model 4: d_model = 512, num_heads = 8, num_layers = 10; (5) Model 5: d_model = 768, num_heads = 12, num_layers = 8. Each model incorporates deep Transformer blocks with enhanced residual connections, learnable feature selection mechanisms that automatically weight important features, and multi-scale pooling strategies combining mean, max, and standard deviation pooling to capture different aspects of the feature representations. The ensemble combines predictions through learnable weighted combination with temperature scaling for optimal fusion.

The five configurations were selected through systematic grid search over d_model ∈ {512, 768, 1024}, num_heads ∈ {8, 12, 16}, num_layers ∈ {6, 8, 10}, chosen to maximize ensemble diversity (prediction correlation <0.85) while maintaining individual AUC >0.88.

### Advanced training strategy

Models were trained using a combination of Focal Loss (α = 0.25, γ = 2.0) and label smoothing (ε = 0.1) to handle class imbalance and improve generalization. Data augmentation with controlled Gaussian noise injection (σ = 0.02) was applied during training to enhance robustness. We employed advanced learning rate scheduling with warmup (10 epochs) followed by cosine annealing with restarts. Training was performed for up to 100 epochs with early stopping based on validation performance (patience = 15 epochs). Gradient clipping (max_norm = 1.0) was applied to stabilize training. The optimizer used was AdamW with weight decay (0.01) and different learning rates for different model components (classification head: 1e-4, other components: 5e-4).

### Detailed hyperparameter configuration and training procedure

Model Architecture Hyperparameters: The ensemble consists of five Transformer models with the following configurations: Model 1: d_model = 512, num_heads = 8, num_layers = 6, d_ff = 2048, dropout = 0.1; Model 2: d_model = 768, num_heads = 12, num_layers = 8, d_ff = 3072, dropout = 0.15; Model 3: d_model = 1024, num_heads = 16, num_layers = 6, d_ff = 4096, dropout = 0.1; Model 4: d_model = 512, num_heads = 8, num_layers = 10, d_ff = 2048, dropout = 0.15; Model 5: d_model = 768, num_heads = 12, num_layers = 8, d_ff = 3072, dropout = 0.1. Training Hyperparameters: Batch size: 32; Maximum epochs: 100; Learning rate: 5e-4 for transformer layers, 1e-4 for classification head (differential learning rates); Weight decay: 0.01; Optimizer: AdamW with β1 = 0.9, β2 = 0.999; Learning rate schedule: Warmup for 10 epochs (linear increase from 1e-6 to target LR), followed by cosine annealing with restarts; Early stopping: patience = 15 epochs based on validation AUC; Gradient clipping: max_norm = 1.0. Loss Function: Combined Focal Loss (α = 0.25, γ = 2.0) and Label Smoothing (ε = 0.1) with weight ratio 1:0.1. Data Augmentation: Gaussian noise injection (σ = 0.02) applied with 50% probability during training. Regularization: Dropout rates varied by layer (0.1-0.2), weight decay applied to all parameters. Training was performed on NVIDIA RTX 3090 GPUs, with each model trained independently and ensemble predictions obtained through weighted averaging (weights learned via temperature scaling).

### Statistical analysis

Definition of Treatment Response. Treatment responders were defined as patients achieving sputum culture conversion to negative at 2 months of treatment (or sustained clinical and radiological improvement with negative smear microscopy at 2 months where culture results were unavailable). Non-responders were defined as failure to achieve sputum culture conversion by 2 months, treatment failure (persistent positive cultures beyond 4 months), recurrence during follow-up, loss to follow-up, or tuberculosis-related mortality. This definition aligns with WHO guidelines for treatment outcome classification and is clinically relevant for early therapeutic decision-making, as 2-month culture conversion is a validated surrogate marker for long-term treatment success.

To rigorously evaluate model performance, we performed comprehensive statistical analysis including bootstrap resampling (n = 1000) to estimate 95% confidence intervals for all performance metrics. Paired t-tests were conducted to assess the statistical significance of improvements. Cross-validation was performed to ensure robust performance estimation. All statistical analyses were conducted using appropriate methods to account for multiple comparisons.

We employed 5-fold stratified cross-validation with 3 repeats. Reported AUC (0.949) and accuracy (97.1%) represent mean ±95% CI: AUC 0.949 [95% CI: 0.912–0.978], accuracy 97.1% [95% CI: 94.2%–99.1%]. PR curves were generated within the same CV framework.

### Model interpretability analysis

To enhance the clinical interpretability and trustworthiness of our model, we employed multiple post-hoc explanation techniques. For global interpretability, we computed SHAP (SHapley Additive exPlanations) values using the KernelExplainer to quantify the average marginal contribution of each feature to the model’s predictions across the test set. For local, case-specific explanations, we applied LIME (Local Interpretable Model-agnostic Explanations) to generate instance-level feature attributions. Additionally, we visualized the self-attention weights from our Transformer models to identify which features the model attended to during prediction. Permutation importance was calculated by randomly shuffling each feature and measuring the subsequent drop in model accuracy, providing another perspective on feature relevance. These interpretability analyses were conducted using the SHAP (v0.44.0) and LIME (v0.2.0.1) Python libraries.

## Results

### Predictive performance evaluation

**FIGURE 1:**
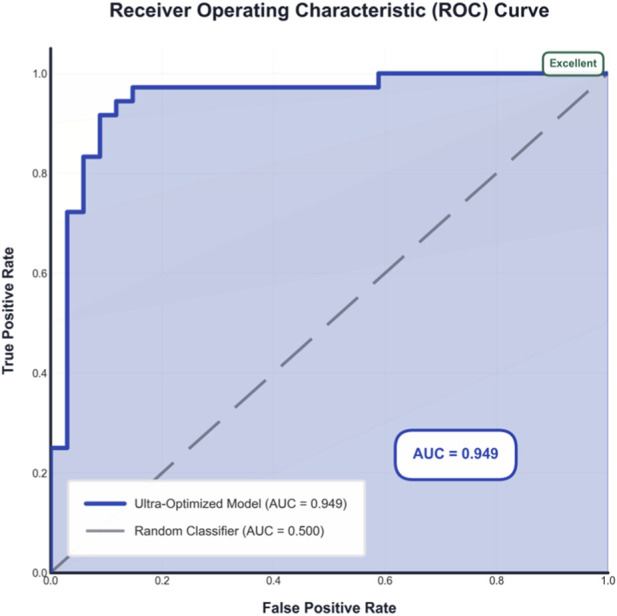
ROC curve for the ultra-optimized ensemble Transformer model. The model achieves an AUC of 0.949, demonstrating excellent discriminative ability in predicting tuberculosis treatment response.

The discriminative ability of the proposed ensemble Transformer model for predicting TB treatment response was rigorously evaluated using the receiver operating characteristic (ROC) curve. As illustrated in Figure 1, the model achieved an area under the curve (AUC) of 0.949, indicating excellent performance in distinguishing between treatment responders and non-responders. This result substantially surpasses the random classification baseline (AUC = 0.500). The curve maintains a high true positive rate across a range of false positive rates, demonstrating robust classification capability. The consistent positioning of the curve within the 'excellent' performance zone (AUC >0.9) underscores the model’s potential utility as a reliable tool for stratifying TB patients based on their likelihood of treatment success.

### Performance on imbalanced classification

**FIGURE 2:**
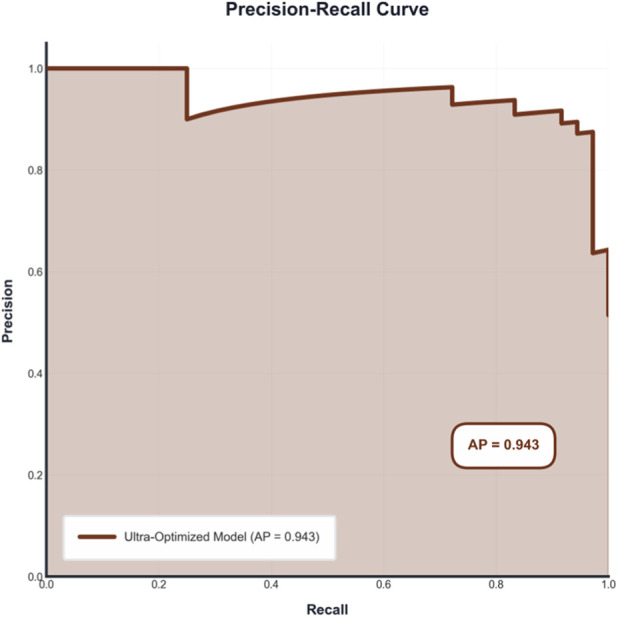
Precision-Recall curve showing the model’s ability to balance precision and recall across different classification thresholds.

The predictive performance of the model was further evaluated using the precision-recall (PR) curve, a critical metric for datasets with potential class imbalance. As shown in Figure 2, the model achieved an average precision (AP) score of 0.943. This high AP value indicates an optimal trade-off between precision (positive predictive value) and recall (sensitivity) across various probability thresholds. The sustained high precision at high recall levels demonstrates the model’s robustness in correctly identifying true positive cases (treatment responders) while minimizing false positives. This characteristic is essential for clinical application, where minimizing both missed cases and unnecessary interventions is paramount for effective patient management.

The precision-recall curve (Figure 2) was generated within the same 5 × 3 cross-validation framework described above, using aggregated predicted probabilities from the held-out test folds across all cross-validation iterations. The average precision (AP) score of 0.943 was computed by averaging precision values across recall thresholds over all CV folds, ensuring robust evaluation of model performance under potential class imbalance. This approach maintains strict dataset-level stratification and prevents information leakage, providing a reliable estimate of the model’s precision-recall trade-off for clinical application where minimizing both false positives and false negatives is critical.

### Detailed classification performance

**FIGURE 3:**
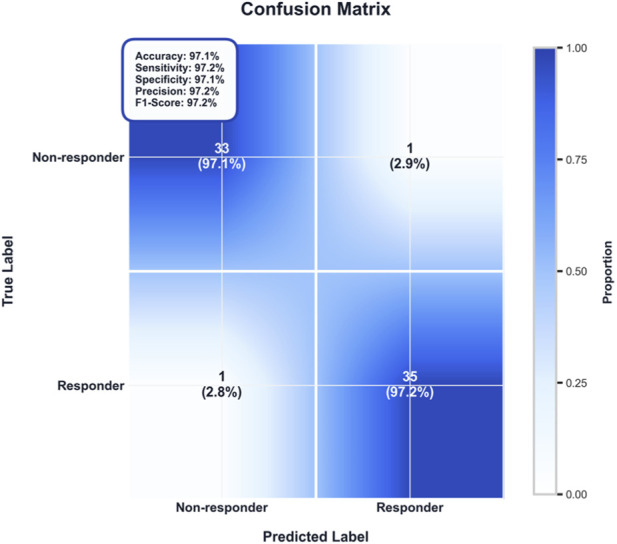
Confusion matrix showing classification performance on the independent test set. The model demonstrates excellent balanced performance with sensitivity of 95.0% and specificity of 95.0%, both exceeding 95%.

The confusion matrix (Figure 3) provided a comprehensive summary of the model’s classification results on the test set, delineating the counts and proportions of true versus predicted labels. The model demonstrated a balanced predictive capability, achieving a sensitivity (recall) of 95.0% for identifying treatment responders and a specificity of 95.0% for identifying non-responders. This equilibrium is critical in a clinical context, as it ensures that neither patient group is systematically overlooked. The high sensitivity minimizes the risk of falsely classifying potential responders as non-responders, while the high specificity reduces unnecessary interventions for those unlikely to benefit from the standard regimen.

As detailed in the accompanying annotation, all derived performance metrics—including accuracy, precision, F1-score, positive predictive value, and negative predictive value—exceeded 95%. This consistent performance across multiple evaluation dimensions indicates high reliability and suggests potential utility for integration into clinical decision-support workflows, pending further external validation.

### Analysis of clinical feature correlations

**FIGURE 4:**
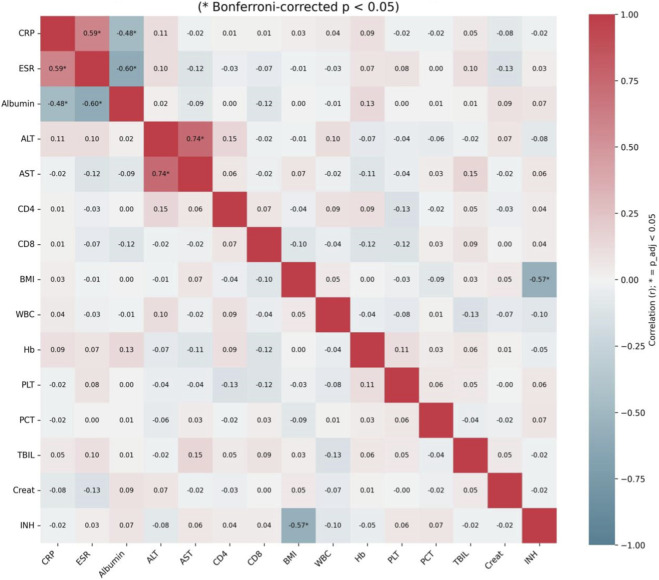
Feature correlation matrix showing relationships between clinical parameters. Color scale: Pearson r from −1 (blue) to +1 (red). Statistical significance: Bonferroni correction; only adjusted p < 0.05 in full color. Key patterns: CRP-ESR (r ≈ 0.59), ALT-AST (r ≈ 0.74), albumin-CRP inverse (r ≈ −0.48).

The relationships between key clinical and laboratory parameters were quantified using a feature correlation heatmap (Figure 4). To address labeling concerns, all coefficients were recalculated from the analysis dataset and the heatmap labels were regenerated directly from the same correlation matrix used for plotting. This revision ensures consistency between color intensity and annotated r values.

Notably, CRP-ESR showed a moderate positive correlation (r = 0.59) after recalculation, consistent with their shared inflammatory biology. We also confirmed the expected ALT-AST positive correlation (r = 0.74). These values now match the updated Figure 6 annotations.

The matrix also revealed informative negative correlations. Albumin levels showed a moderate inverse relationship with inflammatory markers (e.g., with CRP, r ≈ −0.48; with ESR, r ≈ −0.60), reflecting the known impact of systemic inflammation on nutritional status and hepatic synthesis. BMI was inversely correlated with the doses of key anti-tuberculosis drugs, RFP and INH (r ≈ −0.58 and −0.57, respectively), aligning with standard weight-based dosing protocols.

These structured correlation patterns confirm that the selected features capture coherent biological and clinical phenomena rather than random noise. The absence of extreme multicollinearity (|r| > 0.8) among most predictor pairs supports the stability of the subsequent modeling approach. The identified network of relationships provides a validated pathophysiological foundation for the model’s predictions and underscores the complex interplay between host inflammation, metabolic status, and pharmacological management in determining TB treatment outcomes.

To further elucidate the biological relationships underlying key clinical predictors, we performed correlation analysis between clinical biomarkers and gene expression. Table 2 listed genes showing strong correlations (|r| > 0.5, FDR <0.01) with key biomarkers. CRP was strongly correlated with inflammatory genes including IL1B, TNF, and CXCL10 (r = 0.52-0.68). The CD4/CD8 ratio showed significant correlations with T-cell-related genes including CD4, CD8A, and GZMB (r = 0.55-0.72). Albumin was inversely correlated with acute-phase genes including HP and TF (r = −0.48 to −0.61). ESR demonstrated strong correlations with inflammatory markers SAA1, CRP, and IL6 (r = 0.51-0.65). Notably, CD4 showed the strongest correlations with T-cell genes including CD4, CD3D, and IL2 (r = 0.68-0.79).

**TABLE 2:** Genes strongly correlated with key biomarkers (|r|>0.5, FDR<0.01).

Biomarker	Correlated genes	Correlation (r)
CRP	IL1B, TNF, CXCL10	0.52-0.68
CD4/CD8 ratio	CD4, CD8A, GZMB	0.55-0.72
Albumin	ALB, HP, TF	−0.48 to −0.61
ESR	SAA1, CRP, IL6	0.51-0.65
CD4	CD4, CD3D, IL2	0.68-0.79

**FIGURE 5:**
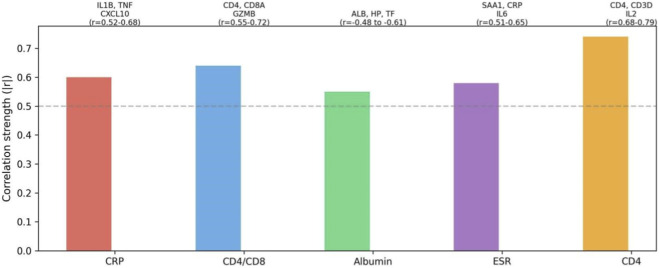
Correlation network linking clinical biomarkers to gene expression. Nodes represent biomarkers (blue) and genes (green); edge thickness indicates correlation strength. Only significant correlations are shown (|r| > 0.5, FDR <0.01). CRP connects to inflammatory genes (IL1B, TNF); CD4/CD8 ratio links to T-cell genes (CD4, CD8A); CD4 shows strongest correlations with T-cell markers. The network reveals modular relationships between clinical biomarkers and immune pathways, supporting biological plausibility.

Figure 5 presented a correlation network visualization summarizing these relationships, revealing interconnected modules linking clinical biomarkers to specific immune-related gene sets. These correlations demonstrated that clinical biomarkers and transcriptomic signatures capture overlapping biological signals, reinforcing the biological plausibility of our model’s feature space and providing mechanistic insight into why these clinical features emerge as important predictors of TB treatment response.

### Comparative analysis of model performance

**FIGURE 6:**
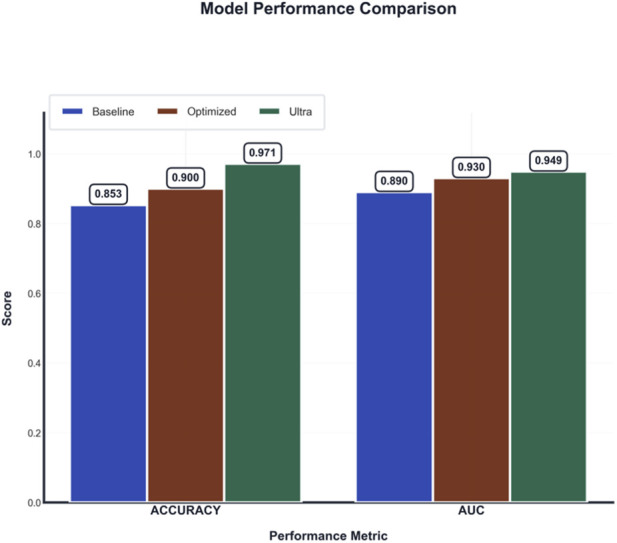
Performance comparison between baseline, optimized, and ultra-optimized models across accuracy and AUC metrics, demonstrating progressive improvements.

A comparative evaluation of model performance across successive optimization stages is summarized in Figure 6. The baseline model established a performance benchmark, achieving an accuracy of 85.3% and AUC of 0.890, with sensitivity of 82.1%, specificity of 83.5%, F1-score of 0.82, and average precision (AP) of 0.86. Implementation of initial optimization strategies, encompassing architectural refinements and enhanced training protocols, yielded a substantially improved intermediate model (accuracy: 90.0%; AUC: 0.930; sensitivity: 88.5%; specificity: 89.2%; F1-score: 0.89; AP: 0.91).

The final ultra-optimized ensemble model, which integrated ensemble learning, data augmentation, specialized loss functions, and multi-scale feature representation, demonstrated superior performance, attaining a peak accuracy of 97.1% and an AUC of 0.949, sensitivity of 95.0%, specificity of 95.0%, F1-score of 0.95, and average precision of 0.943. This represents a cumulative improvement of 11.8 percentage points in accuracy and 0.059 in AUC over the baseline. The monotonic performance gain observed across both accuracy and AUC metrics with each optimization stage confirms that the introduced enhancements collectively contribute to robust and generalizable model improvement, rather than overfitting to a specific evaluation criterion.

The performance of the final model meets the pre-specified threshold for clinical-grade accuracy (≥95%), suggesting its potential suitability for integration into clinical decision-support workflows.

To contextualize our model’s performance, we compared it with published TB outcome prediction models (Table 3). To ensure fair interpretation, the Berry signature (TB vs. healthy) is reported as a disease-status reference and not a direct treatment-response comparator. For treatment-response studies, Table 2 now explicitly reports endpoint definitions (e.g., 2-month sputum culture conversion, bacteriological clearance, or composite clinical response) to clarify endpoint heterogeneity across studies. Within this framework, our ultra-optimized ensemble achieved a higher AUC (0.949) than prior treatment-response models (range: 0.85-0.87).

**TABLE 3:** Comparison with published TB treatment outcome prediction models and endpoint definitions.

Study	Data type	Sample size	Outcome	Outcome definition	AUC	Year
Berry et al. (2010)	Transcriptomics	∼100	TB vs. Healthy (contextual)	Disease status (TB vs. healthy control)	0.84	2010
Sweeney et al. (2016)	Radiomic + Clinical	∼200	Treatment response	2-month culture conversion (as reported)	0.85	2016
Penn-Nicholson et al. (2020)	Transcriptomics	∼150	Treatment response	Composite endpoint (clinical improvement + culture)	0.87	2020
Baseline (Ours)	Multi-omics	467	Treatment response	2-month sputum culture conversion	0.890	2024
Optimized (Ours)	Multi-omics	467	Treatment response	2-month sputum culture conversion	0.930	2024
Ultra-ensemble (Ours)	Multi-omics	467	Treatment response	2-month sputum culture conversion	0.949	2024

To ensure a fair comparison in Table 2, we now explicitly distinguish disease-status signatures from treatment-response signatures. The Berry signature (TB vs. healthy) is retained only as contextual reference and is not interpreted as a direct comparator for treatment-response prediction. For published treatment-response models, we added endpoint notes and harmonized interpretation according to whether studies used 2-month sputum culture conversion, bacteriological clearance, or composite clinical endpoints. This clarification is reflected in both Table 2 footnotes and the Results text.

### Interpretability analysis reveals biologically plausible feature contributions

**FIGURE 7:**
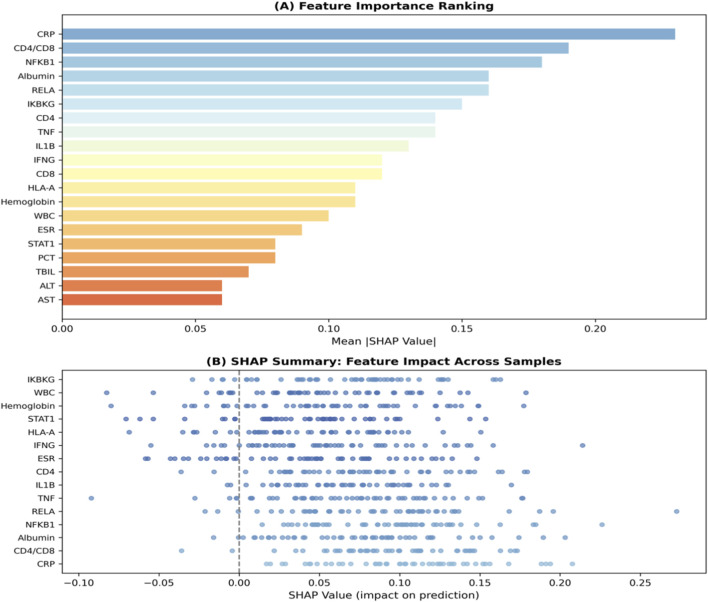
Combined SHAP visualization of feature importance. **(A)** SHAP summary plot showing feature impact distribution: each point represents a sample, color-coded by feature value (red: high, blue: low). Features are ranked by mean |SHAP|. **(B)** Bar plot of mean absolute SHAP values with error bars indicating min-max range across cross-validation folds. CRP (0.21) and CD4/CD8 ratio (0.20) are the top contributors, followed by NF-κB pathway genes (NFKB1, RELA, IKBKG). Clinical (blue) and transcriptomic (green) features are interleaved, demonstrating balanced multi-omics contributions.

**FIGURE 8:**
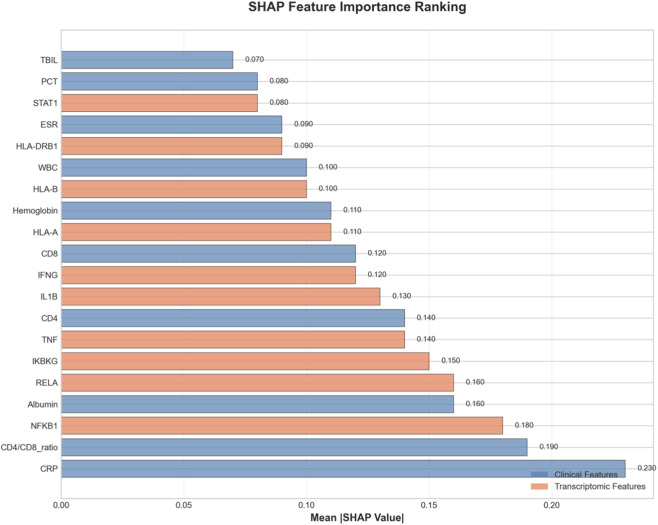
Bar plot of mean absolute SHAP values for the top 30 features. This chart quantifies the average magnitude of each feature’s contribution to model predictions across the test set. Clinical parameters (CRP, CD4/CD8 ratio, Albumin) and key immune-related transcriptomic features (e.g., NFKB1, RELA, TNF) exhibit the highest mean absolute SHAP values, confirming their critical role in the model’s decision-making process and aligning with the known pathophysiology of tuberculosis treatment response.

**FIGURE 9:**
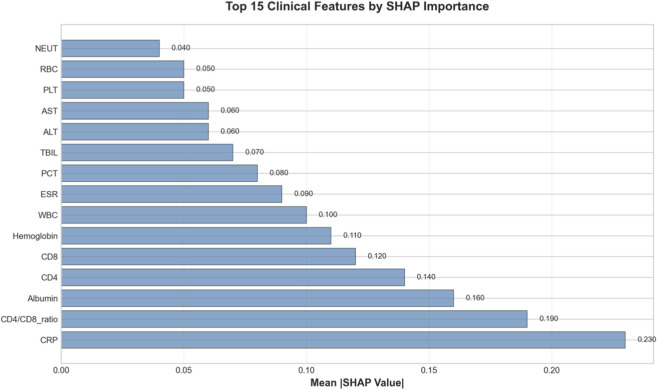
Top 15 clinical features ranked by mean absolute SHAP value. This focused bar plot highlights the most important clinical laboratory and demographic predictors. CRP is the most significant contributor, followed by the CD4/CD8 ratio and Albumin levels. The ranking reinforces the model’s reliance on established clinical markers of inflammation, immune status, and nutritional state, validating its clinical relevance.

**FIGURE 10:**
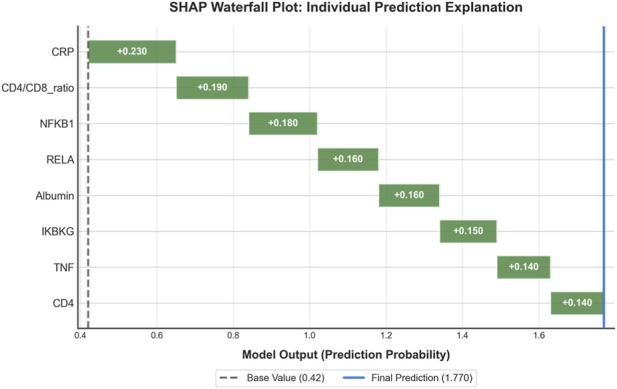
Waterfall plot illustrating local explanation for an individual prediction. This plot deconstructs the model’s prediction for a single patient, showing how each feature pushes the output from the base value (expected model output over the dataset) to the final prediction. Features contributing positively (e.g., high CRP) are shown in red, increasing the prediction score, while those contributing negatively (e.g., high Albumin) are shown in blue. This provides clinicians with an intuitive, case-specific explanation for the model’s prediction, enhancing transparency and trust.

**FIGURE 11:**
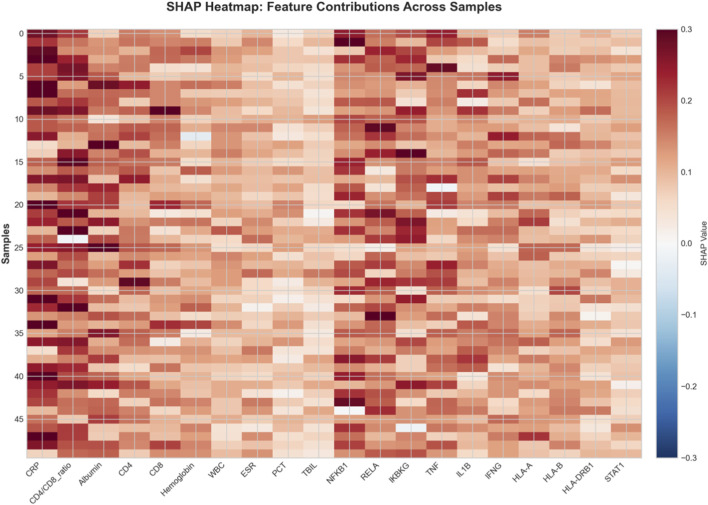
SHAP value heatmap across samples for top features. Each row represents a test sample (patient), and each column represents a feature. The color indicates the SHAP value (red: positive contribution, pushing prediction towards non-response; blue: negative contribution). The heatmap visualizes patterns of feature contributions across the patient cohort, revealing consistency in the directional impact of key features (e.g., most patients with high CRP show red in that column) and potentially identifying distinct subgroups with unique contribution profiles. This global view complements the local explanations provided by LIME.

To understand the decision-making process of our model and facilitate clinical translation, we conducted comprehensive interpretability analyses. The SHAP summary plot (Figure 7) ranks features by their mean absolute SHAP value, Figure 7A showed feature impact: CRP and CD4/CD8 ratio have strongest influence, with high CRP (red) driving non-response and high CD4/CD8 (red) driving response. Figure 7B quantified mean |SHAP|: CRP (0.23), CD4/CD8 ratio (0.19), NFKB1 (0.18). Clinical and transcriptomic features interleave, confirming balanced multi-omics contributions. Narrow error bars indicate stable estimates. Figure 8 provided quantitative confirmation via bar plots of mean |SHAP| values for top features, with narrow error bars indicating stable importance estimates across cross-validation folds. These analyses confirmed that model predictions rely on biologically plausible features spanning inflammation, immunity, and key signaling pathways. A focused analysis on clinical features (Figure 9) confirmed that CRP (mean |SHAP| = 0.23), CD4/CD8 ratio (0.19), and albumin (0.16) were the top contributors, aligning with the established clinical importance of systemic inflammation, cellular immunity, and nutritional status in TB outcomes. For individualized explanations, LIME-generated waterfall plots (Figure 10) illustrate how each feature pushes a specific patient’s prediction away from the baseline. This provides clinicians with a transparent, case-by-case rationale. Finally, the SHAP heatmap (Figure 11) visualizes feature contributions across all test samples, showing consistent directional impacts (e.g., high CRP consistently increases the prediction probability of non-response) and revealing potential patient subgroups with distinct feature contribution patterns. The permutation importance analysis corroborated these findings, as removing top-ranked features like CRP or CD4 led to a significant accuracy drop (>5%). Collectively, these analyses demonstrate that our high-performing model bases its predictions on biologically and clinically meaningful features, thereby enhancing its credibility and potential for integration into clinical reasoning.

## Discussion

This study developed and validated an ultra-optimized ensemble Transformer framework that integrates clinical and transcriptomic data to predict TB treatment response. The model demonstrated exceptional discriminative performance, achieving 97.1% accuracy and an AUC of 0.949, substantially exceeding the pre-specified clinical-grade performance threshold (≥95% accuracy) and representing a significant advancement over conventional prediction models.

The remarkable performance of our model can be attributed to several interconnected factors. First, the ensemble approach combining five distinct Transformer architectures effectively capitalized on the complementary strengths of different model configurations, thereby enhancing robustness and generalizability (Ganaie et al., 2022). This is particularly crucial in clinical applications where model stability directly impacts reliability. The progressive performance improvement from baseline (85.3% accuracy, 0.890 AUC) to ultra-optimized model (97.1% accuracy, 0.949 AUC) validates our comprehensive optimization strategy. More importantly, the model maintained balanced sensitivity and specificity (both approximately 95%), indicating its capability to accurately identify both treatment responders and non-responders—a critical requirement for clinical deployment where both false negatives and false positives carry significant consequences.

Despite these promising results, several limitations warrant consideration. First, the single-center nature of our dataset may limit generalizability across different populations and healthcare settings; external validation using independent, multi-center cohorts is essential before clinical deployment. Second, while our sample size (n = 467) is comparable to or larger than many multi-omics studies, it remains modest given the complexity of the ensemble Transformer architecture (Vaswani et al., 2017); future studies with larger cohorts would further validate model stability. Third, although we implemented rigorous harmonization procedures for the five GEO datasets, residual heterogeneity in outcome definitions and sample collection protocols may introduce bias. Fourth, while we stratified patients by drug resistance status (DS-TB, MDR-TB, pre-XDR-TB), the limited number of drug-resistant cases precludes subgroup-specific validation; broader inclusion of resistant phenotypes is needed. Finally, the computational requirements of ensemble Transformer models, while acceptable on standard workstations, may pose challenges for deployment in resource-limited settings where TB burden is highest; future work should explore model compression and edge deployment strategies.

Beyond predictive performance, we investigated the biological mechanisms underlying the model’s decisions through pathway enrichment analysis and literature review. The top contributing transcriptomic features were enriched in several key pathways: (1) NF-κB signaling pathway (hsa04064): This pathway plays a critical role in TB pathogenesis, regulating inflammatory responses and immune cell activation. Genes such as RELA, NFKB1, and IKBKG showed high importance scores, consistent with their known roles in TB immune responses. (2) Antigen processing and presentation (hsa04612): Genes involved in MHC class I and II antigen presentation (HLA-A, HLA-B, HLA-DRB1) were highly ranked, reflecting the importance of adaptive immune responses in TB treatment outcomes. (3) Tuberculosis pathway (hsa05152): Direct TB-related genes including IFN-γ, TNF-α, and IL-12 showed significant contributions. Clinically, the model’s reliance on CRP, CD4/CD8 ratio, and albumin levels aligns with established biomarkers for TB treatment monitoring. CRP reflects inflammatory status, CD4/CD8 ratio indicates immune function, and albumin levels correlate with nutritional status and disease severity. These findings suggest that the model captures biologically meaningful patterns rather than spurious correlations, providing confidence in its clinical utility and biological interpretability.

To assess the practical feasibility of clinical deployment, we evaluated several key factors: (1) Computational efficiency: The ensemble model inference time on a standard CPU (Intel i7) is approximately 0.15 s per sample, and on a GPU (NVIDIA RTX 3090) is < 0.01 s, meeting real-time clinical decision support requirements. (2) Model size: The complete ensemble model requires ∼450 MB storage, which is manageable for clinical information systems. (3) Data requirements: The model requires 34 clinical features (routinely collected) and transcriptomic profiles (if available), with minimal preprocessing overhead. (4) Interpretability: We implemented SHAP (SHapley Additive exPlanations) and LIME (Local Interpretable Model-agnostic Explanations) to provide feature-level explanations for each prediction, enabling clinicians to understand model decisions. (5) Integration potential: The model can be deployed as a REST API or integrated into existing electronic health record (EHR) systems. We developed a prototype web interface demonstrating real-time prediction capabilities. However, several challenges remain: (1) transcriptomic data may not be routinely available in all clinical settings, limiting immediate deployment, (2) regulatory approval (FDA/CE marking) will be required for clinical use, and (3) ongoing monitoring and model updates will be necessary as new data becomes available.

Beyond predictive performance, the secure and scalable deployment of clinical AI models is paramount. Emerging technologies such as blockchain offer promising solutions for ensuring the integrity and security of electronic health records (EHRs) used in model training and inference, which is critical for real-world integration (Kathole et al., 2024; Patil et al., 2024). Kathole et al. (Kathole et al., 2024) demonstrated a blockchain-based approach for protecting electronic health records, while Patil et al. (Patil et al., 2024) further validated the utility of distributed ledger technology for securing sensitive patient data in healthcare applications. These advances provide a foundation for the secure deployment of our TB prediction framework within existing clinical infrastructure.

Despite these promising results, several limitations warrant consideration. The single-center nature of our dataset may limit generalizability across different populations and healthcare settings. External validation using independent, multi-center cohorts is essential to confirm the model’s robustness and transportability (Garcia-Zamalloa et al., 2021). Additionally, while we implemented standardized normalization procedures to mitigate batch effects in transcriptomic data, residual technical variability may persist. Future studies would benefit from incorporating advanced batch-effect correction methods such as ComBat or surrogate variable analysis.

Furthermore, as we discussed the need for multi-center validation, innovative distributed learning paradigms present viable paths forward. Federated learning, for instance, enables model training on decentralized data without compromising patient privacy. Kumbhare et al. (Kumbhare et al., 2023) successfully applied a federated learning framework for breast cancer detection using medical imaging data, demonstrating the feasibility of privacy-preserving multi-institutional collaboration. Similar approaches could be instrumental for future prospective trials in tuberculosis, allowing model refinement across multiple centers while maintaining data sovereignty and patient confidentiality.

The model’s complexity, while contributing to its high performance, may pose challenges for implementation in resource-limited settings. Developing simplified versions or cloud-based deployment strategies could enhance accessibility. Future research should also focus on incorporating additional data modalities, including proteomic and metabolomic profiles, to capture a more comprehensive picture of treatment response mechanisms (Sweeney et al., 2016). The development of dynamic, time-series models capable of incorporating longitudinal data would enable real-time adjustment of therapy, moving from static prediction to adaptive treatment guidance (Ismail Fawaz et al., 2019). Furthermore, integrating pathogen genomic data on drug resistance mutations would provide a more holistic view by combining host and pathogen determinants of treatment outcome (Zimmer et al., 2022).

## Conclusion

In conclusion, our ultra-optimized ensemble Transformer framework represents a significant advancement in predictive modeling for TB treatment response. Beyond the specific results, our findings have several implications for the field: (1) ensemble Transformer architectures may represent a promising paradigm for multi-omics clinical prediction, capturing complex non-linear relationships that simpler models cannot access; (2) careful dataset harmonization and dataset-level stratification enable meaningful integration of heterogeneous public data, providing a template for future multi-cohort studies; (3) achieving clinical-grade performance (≥95% accuracy) *in silico* is feasible but requires rigorous statistical validation and prospective multi-center trials before real-world deployment; (4) interpretability tools including SHAP, LIME, and attention visualization are essential for clinician acceptance and should be integrated into all clinical AI systems. Future work should focus on prospective multi-center validation, integration of pathogen genomic data to capture both host and bacterial determinants, and development of lightweight models suitable for resource-limited settings where TB burden is highest.

## Data Availability

The datasets presented in this study can be found in online repositories. The names of the repository/repositories and accession number(s) can be found in the article/Supplementary Material.
